# Letter to the Editor Regarding: Evolving Business Models in Orthotics

**DOI:** 10.33137/cpoj.v5i1.37717

**Published:** 2022-07-17

**Authors:** L. Laakso

**Affiliations:** 1 Orthotics Prosthetics Canada (OPC), Toronto, Canada.; 2 Custom Orthotic Design Group Ltd., Mississauga, Canada.

Dear Canadian Prosthetics & Orthotics Journal Editorial Board,

I am the President of Orthotics Prosthetics Canada (OPC), the credentialing body for orthotic and prosthetic professionals in Canada. OPC exists for the fundamental purpose of assisting patients in maintaining functional and productive lives by setting standards of education, credentialing and practice for the healthcare professionals who provide orthotic and prosthetic care. The goals of OPC are like that of the Canadian Prosthetics and Orthotics Journal (CPOJ): your, “*passion for promoting and disseminating knowledge*” is consistent with our role of, “*advancing the profession of orthotics and prosthetics through quality standards of practice, professional credentialing, education and awareness*”.

The purpose of this letter is to initiate a constructive dialogue, clarify some of the information provided in the CPOJ recent article,^1^ “*Evolving business models in orthotics*” and to highlight our concerns with respect to important context about the orthotic and prosthetic profession in Canada that we feel was missing from the publication.

The article^1^ states, “*Certification is limited to public education*”. I will note that the pathway to becoming credentialed in Canada includes an undergraduate degree in engineering, kinesiology, or related program prior to a two-year certificate program at an OPC accredited orthotic and prosthetic school. After completion of the formal education program, candidates (referred to as Residents) must complete a 3,450-hour residency and successfully complete the OPC certification examinations. The policies, procedures, school curricula, educational objectives, and the examination themselves are based on a validated practice analysis and evidence-based examination methodologies. Based on these facts, we hope it is clear that the process to become certified in Canada is much more comprehensive than “public education”.

The pathway to becoming a Certified Orthotist or Certified Prosthetist in Canada is accredited by the International Society of Prosthetics and Orthotics (ISPO) which is the standard of reference for the World Health Organization (WHO) for prosthetic orthotic occupations. Although the profession is not licensed provincially, due to several factors that include but are not limited to cost, it is regulated by OPC and therefore recognized by many of the provincial health ministries, including Alberta, the province under scrutiny. Regulation is identified as a core pillar within the scope of OPC, whose mandate is, “*to protect the public and advance the profession of orthotics and prosthetics through quality standards of practice, professional credentialing, education and awareness*”. Further, professional credentialing makes up another core pillar of OPC, which is validated through the external parties of the ISPO, the WHO, several provincial ministries of health and federal healthcare programs.

Certification of the profession in Canada is recognized globally and is one of six entities that employ nine core practitioner standards including minimum education standards, entry level competency standards, scope of practice, code of ethics, course accreditation, continuing profession education, language standards, recency of practice and return to practice standards within the orthotics and prosthetics profession worldwide.^2^ The WHO has stated, in the document, Prosthetics for Orthotics Standards & Implementation Guide,^3^ Standard 25, “*Prosthetics and orthotics services should be provided by competent, adequately trained professionals.*” OPC Certified members are recognized and credentialled by ISPO and therefore recognized by the WHO as the trained orthotic and prosthetic professionals in Canada. The Standards within the guide are part of the requirements for Canada to fulfill in order to meet our obligations under the Convention on the Rights of Persons with Disabilities (CRPD).

To clarify the comments regarding number of practitioners we have in Canada, I would like to share the following information:

1. OPC has identified and acknowledged a potential shortage in the number of practitioners in the future and is addressing the issue through our National Education Standards Project. We are working to establish more education opportunities based on demographic data. In fact, two additional students are enrolled within the current programs in the 2021-2022 school year cohort. If Alberta, or any other jurisdiction has recognized a shortage of practitioners, OPC would welcome the opportunity to assist in the creation and facilitate the development of an accredited orthotics and prosthetics program at one of the universities in Alberta or local province.
2. Since 2010, we have seen 18% growth in the number of practitioners in Canada and we strive to continue to work towards the goal set by the WHO of 15-20 prosthetists/orthotists per million people.^4^

A final Call to Action from the article is to recognize people who are trained in orthotics and prosthetics from outside of Canada. To be clear, the role of OPC is to protect the public in Canada and recognizes that the standards of practices from different countries are not the same as it is in Canada. OPC provides a pathway for foreign trained applicants, including those who have been recognized by ISPO. It is important to note however, that ISPO sets standards for education that are global and do not reflect the specific scope of practices of various countries or jurisdictions. ISPO education standards are setting specific and are vetted by experts in each of those settings for schools to become accredited. In that respect, although ISPO sets a minimum standard for *education*, it is not always equal to the minimum standard of *practice* in orthotics and prosthetics in Canada. Therefore, there may be variance in training pathways and competencies of graduates that require assessment specific to the context in Canada.

Orthotics and Prosthetics Canada has in place an objective and transparent assessment for graduates from foreign trained pathways and programs. By having all practitioners achieve a minimum standard for entry to practice as developed based on a practice analysis within Canada, we are creating a consistent standard that reflects the needs and expectations of patients and healthcare professionals within our country. Many health care professions including the Medical Council of Canada and the Canadian Physiotherapy Association have similar practices with respect to people who are educated and/or trained outside of our borders.

I hope that this has provided some clarity with respect to the stakeholder perspective article^1^that you have published. If you, or any of your readers require more information or clarification, please do not hesitate to contact us.

## DECLARATION OF CONFLICTING INTERESTS

I have the following conflicts of interest to declare: I am President, Orthotics Prosthetics Canada (OPC), the Past President, International Society of Prosthetics and Orthotics Canada (ISPO Canada) and I am an Owner, Practitioner of the Custom Orthotic Design Group Ltd. in Mississauga, Canada.

## References

[1] Schneider N. Evolving business models in orthotics. Canadian prosthetics & orthotics journal. 2021; Volume 4, Issue 2, No.3. 10.33137/cpoj.v4i2.35876 PMC10443506 37614999

[2] Clarke L, Puli L, Ridgewell E, Dillon MP, Anderson S. Regulation of the global orthotist/prosthetist workforce, and what we might learn from allied health professions with international-level regulatory support: a narrative review. Hum. Resour. Health. 2021 Dec;19(1):1–4. DOI: 10.1186/s12960-021-00625-9 34266431 PMC8281620

[3] Standards for prosthetics and orthotics, part 1: standards [Internet]. World Health Organization. [cited 2021 October 22]. Available from: https://apps.who.int/iris/bitstream/handle/10665/259209/9789241512480-part1-eng.pdf?sequence=1&isAllowed=y

[4] Standards for prosthetics and orthotics, part 2: implementation manual [Internet]. World Health Organization. [cited 2021 October 22]. Available from: https://apps.who.int/iris/bitstream/handle/10665/259209/9789241512480-part2-eng.pdf?sequence=2&isAllowed=y

